# Robot-Assisted Colorectal Cancer Surgery Mitigates Early Postoperative Immunosuppression and Angiogenesis

**DOI:** 10.3390/ijms262010041

**Published:** 2025-10-15

**Authors:** Mariusz G. Fleszar, Marek Zawadzki, Paulina Fortuna, Iwona Bednarz-Misa, Izabela Krauze, Kamila Maciejewska, Jakub Klekowski, Mariusz Chabowski, Wojciech Witkiewicz, Małgorzata Krzystek-Korpacka

**Affiliations:** 1 Omics Research Center, Wroclaw Medical University, 50-368 Wroclaw, Poland; paulina.fortuna@umw.edu.pl; 2 Department of Oncological Surgery, Regional Specialist Hospital, 51-124 Wroclaw, Poland; zawadzki@wssk.wroc.pl (M.Z.); witkiewicz@wssk.wroc.pl (W.W.); 3 Department of Clinical Surgical Sciences, Faculty of Medicine, Wroclaw University of Science and Technology, 50-556 Wroclaw, Poland; mariusz.chabowski@gmail.com; 4 Department of Biochemistry and Immunochemistry, Wroclaw Medical University, 50-368 Wroclaw, Poland; iwona.bednarz-misa@umw.edu.pl (I.B.-M.); izabela.krauze@umw.edu.pl (I.K.); kamila.maciejewska@umw.edu.pl (K.M.); 5 Department of Nursing and Obstetrics, Division of Anesthesiological and Surgical Nursing, Faculty of Health Science, Wroclaw Medical University, 50-367 Wroclaw, Poland; 6 Department of Surgery, 4th Military Clinical Hospital, 50-981 Wroclaw, Poland; 7 Research and Development Centre at Regional Specialist Hospital, 51-124 Wroclaw, Poland

**Keywords:** surgical stress, minimally invasive surgery, growth factors, angiogenesis, Th1 and Th2 immunity, prostaglandin, postoperative immunosuppression, systemic inflammatory response, cytokine storm, immunocompetence

## Abstract

Minimally invasive surgery is known to lessen postoperative stress and complications compared with open procedures, yet its molecular effects on immunity and cancer-related mechanisms remain unclear. This study examined immune and inflammatory responses after robot-assisted (RS) versus open (OS) colorectal cancer surgery. Sixty-one patients (RS = 30; OS = 31) were enrolled. Blood samples were collected before surgery and at 8, 24, and 72 h post-incision. Cytokines, growth factors, and prostanoids were measured using multiplex immunoassays and mass spectrometry to assess systemic immune and inflammatory changes. Surgery type markedly influenced perioperative immune profiles. RS induced stronger activation of Th1-associated cytokines, including IFNγ and IP-10, suggesting enhanced cellular immune responsiveness. In contrast, Th2 cytokines and other immunosuppressive mediators—such as IL-4, IL-10, and G-CSF—showed smaller or transient increases after RS, whereas OS triggered broader and more sustained elevations. Angiogenic factors (VEGF-A, PDGF-BB, FGF2) rose significantly after OS but remained comparatively lower and returned to baseline faster after RS, indicating a weaker proangiogenic response. Similarly, postoperative surges in prostaglandins linked to inflammation and tumor progression (PGE_2_, PGF_2_α) were blunted and resolved earlier following RS. Overall, the robotic approach was associated with reduced inflammatory and immunosuppressive activity, faster recovery of immune balance, and diminished biochemical signals favoring angiogenesis and potential tumor regrowth, suggesting a potential protective effect against pathogens and cancer-promoting mechanisms after colorectal tumor resection.

## 1. Introduction

Colorectal cancer (CRC) ranks third in incidence and second in mortality worldwide [1,2]. Its incidence and mortality doubled from 1990 to 2021, rising to 2.19 and 1.04 million cases, with further growth anticipated [3]. While remaining the highest, CRC incidence in high-income countries is gradually declining due to nationwide screening programs [1,2]. In contrast, low- and middle-income countries face rising incidence and persistently high mortality, driven by Westernized lifestyles and limited access to diagnostics and therapy [1,3,4,5]. Europe, with less than 10% of the world’s population, accounts for nearly 30% of global CRC cases [2], and Poland reports the highest age-standardized mortality among the five most populous European nations [6,7]. Although CRC remains a major burden among the elderly [8], incidence is rapidly increasing in younger adults (<50 years) [5,9,10], regardless of their country’s income [10]. Early-onset CRC incidence is higher in males and projected to rise further, in contrast to the slight decline expected for females [5,9]. This sex-related disparity is most pronounced in high-sociodemographic regions and extends to survival outcomes [9].

Surgical resection remains the cornerstone of curative treatment for CRC—not only in early-stage and localized disease but also in selected cases of metastatic cancer, particularly when secondary liver or lung lesions are resectable [11]. Robot-assisted surgery (RS), the most advanced form of minimally invasive surgery, offers notable advantages over open surgery (OS) [12] and conventional laparoscopy (LS) [13,14,15,16,17,18,19,20]. These include superior preservation of critical structures such as nerves and blood or lymphatic vessels, enhanced precision due to tremor filtration, improved articulation and ergonomics, and high-definition, three-dimensional visualization provided by a stable, self-controlled camera [14,19,21]. Clinically, RS has been associated with reduced rates of both minor [20,22,23] and major complications [14,16], fewer conversions to open procedures [14,16,17,18,24], more thorough lymphadenectomy [14,15,19,20], less postoperative pain [20], and faster recovery—measured by earlier catheter and drain removal, earlier return to diet, and quicker restoration of bowel function—all contributing to shorter hospital stays [13,14,20]. Importantly, RS offers shorter hospitalization and lower complication rates, including conversions to OS, than LS also in an emergency surgery setting, where RS selection rose from none to 20% in 2025 [25].

Short-term benefits, including improved 30-day survival [26], have been supported by meta-analyses [21,26,27,28]. Evidence regarding long-term oncologic superiority remains inconclusive [26], although more recent data are in favor of improved outcomes [29], including lower recurrence rates [30]. While RS may reduce tissue trauma [14,16,17,18,22], it also presents challenges such as prolonged operative time, increased exposure to general anesthesia, higher costs, absence of tactile feedback, and the need for specialized training [12,28]. Whether the clinical benefits justify the financial burden of RS remains an ongoing debate, with arguments both in favor [14,28] and against [19,31,32].

As emphasized by Dobson [33], the impact of surgical stress on recovery and oncologic outcomes remains underappreciated—often considered the “neglected step-child of global health”. The physiological stress response to major surgery is a survival mechanism initiated by an inflammatory cascade known as the systemic inflammatory response (SIR). This response activates innate immunity, primarily involving neutrophils, and is aimed at preventing infection, preserving immune competence, clearing cellular debris, and facilitating tissue repair and regeneration [34,35]. SIR is typically followed by the compensatory anti-inflammatory response (CAR), mediated by lymphocyte-driven suppression of adaptive immunity [34,35].

When these responses become dysregulated, pathological conditions may arise. Hyperinflammation (SIR syndrome, SIRS) can lead to multi-organ failure, whereas excessive immunosuppression (CAR syndrome, CARS) increases the risk of secondary infections and, in cancer patients, tumor immune escape [34,36]. Maintaining a balanced SIR–CAR interplay is crucial for individuals with malignancy, whose immune systems are compromised even prior to surgery. Furthermore, the Th2-dominant, immunosuppressive environment seen in cancer is exacerbated by surgical trauma [37,38]. Hence, neuroendocrine, metabolic, immune–inflammatory, and regenerative processes must be precisely regulated. These are orchestrated by a network of primary messengers, including hormones, cytokines, growth factors, and prostanoids. Disruption of this delicate balance can result in excessive oxidative stress and tissue damage, impaired healing, and weakened immune surveillance—factors that may increase the risk of metastasis or cancer recurrence, which affects up to 30% of CRC patients within five years of treatment [39,40,41,42].

Considering the above, the dynamics of immune mediators in the early postoperative period are likely to quantify physiological stress better than subjective measures. Their monitoring allows for comparison of the extent of post-surgical immune suppression, which can affect tumor surveillance and recurrence, and the speed of immune recovery, which relates to wound healing and infection risk. The degree of tissue trauma correlates with the magnitude of stress response; therefore, RS, as a minimally invasive procedure, may reduce immune perturbation compared with OS. While LS has indeed been shown to attenuate the postoperative stress response, data on the immunological and molecular impact of RS remain limited. In pioneering work, Shibata et al. [43] demonstrated improved markers of immunocompetence (HLA-DR expression) following RS but not LS. Our group has previously shown that RS, compared to OS, attenuates lymphopenia [44], reduces cortisol peaks [45] and inflammatory mediator levels [46], modulates chemokine release [47], preserves brominated tyrosine [48], and restores the immunonutrient arginine [49]. These findings suggest a dampened systemic stress response following RS.

Building upon this foundation, the present study aims to comprehensively assess the postoperative (0–72 h) immune–inflammatory and growth-factor responses in CRC patients undergoing RS versus OS. Specifically, we analyzed Th1-associated cytokines (IL-12p70, IL-17A, IFN-γ, IP-10) and Th2-associated cytokines (IL-4, IL-5, IL-9, IL-10, IL-13), as well as growth factors (VEGF-A, FGF-2, PDGF-AA, G-CSF, GM-CSF) and prostanoids (PGE_1_, PGE_2_, PGF_1_α, PGF_2_α, PGD_2_, PGJ_2_, TXB_2_, LTB_4_) involved in inflammation, immune regulation, tissue repair, and angiogenesis. By identifying molecular differences in early postoperative stress responses, we aim to better understand the potential of RS to influence recovery trajectories and long-term oncologic outcomes.

## 2. Results

Representatives of Th1/Th2 immunity, proangiogenic growth factors and colony-stimulating factors as well as prostanoids–lipid mediators of inflammation, immunity, and angiogenesis were measured using Luminex xMAP^®^ technology and mass spectrometry in 61 CRC patients. Their perioperative dynamics were compared between patients undergoing open or robotic procedures, well matched with respect to age, sex, general health, and the disease advancement, to discern potential differences with respect to surgery type.

### 2.1. Immune-Respone Mediators

#### 2.1.1. Inducers and Mediators of Th1 Response

**Figure 1:**
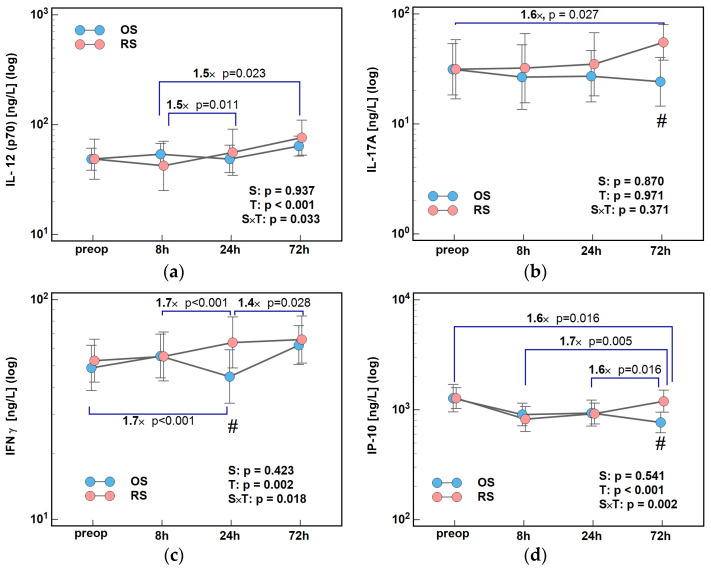
The effect of surgery type on changes in Th1 cytokine concentrations during the early postoperative period: (**a**) IL-12 (p70); (**b**) IL-17A; (**c**) IFNγ; and (**d**) IP-10. Data were analyzed using repeated measures of analysis of variance (ANOVA) and the results are presented as probabilities (p) of effect significance for surgery (S) and time (T), and their interaction (S × T). Data are presented as geometric means with 95% CI (dots with whiskers) with significant differences in cytokine concentration at a given time-point marked by hash (#) and differences in relative change between time-points (ratios) marked by connectors with magnitude of difference. OS, open surgery; RS, robotic surgery; preop, preoperative cytokine concentration; and CI, confidence interval.

Time of blood collection significantly affected concentrations of IL-12(p70), IFNγ, and IP-10 (*p* value for time (T) factor ≤ 0.05) but not IL-17A. Surgical approach significantly affected concentration changes over time of IL-12(p70), IFNγ, and IP-10, which is indicated by statistically significant interaction factor S × T (surgery × time), but had no impact on IL-17A time-course (Figure 1). Still, changes in IL-17A concentrations over time were directed upward in RS and downward in OS, the most dynamically between 24 and 72 h, yielding 72/0 h ratios 1.6× higher in RS (Figure 1b).

IL-12(p70) slightly peaked at 8 h and then rose between 24 h and 72 h in OS but dropped initially in RS and then increased until 72 h. Consequently, 24/8 h and 72/8 h ratios were significantly higher, by 1.5×, in RS (Figure 1a).

Concentrations of IFNγ were steadily increasing during follow-up after RS but dropped at 24 h following OS, so that both groups differed with respect to their 24/0 h (by 1.7×), 24/8 h (by 1.7×), and 72/24 h (by 1.4×) ratios (Figure 1c).

IP-10 dropped initially in both groups but then was rising in RS while decreasing in OS, so that their 72/0 h and 72/8 h ratios were significantly higher in RS by 1.6× and 72/24 h by 1.7× (Figure 1d).

**Figure 2:**
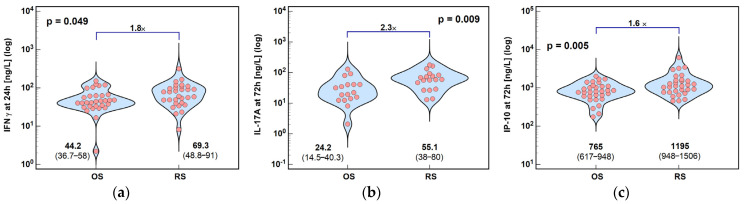
Effect of surgery type on concentrations of cytokines: (**a**) IFNγ at 24 h; (**b**) IL-17A at 72 h; and (**c**) IP-10 at 72 h post-incision. Data were analyzed using *t*-test for independent samples or Mann–Whitney *U* test. Test results are presented as means or medians, respectively, with 95% CI and probability *p*. The magnitude of between-group differences is indicated by numbers above connectors. Data distribution is illustrated by violin plots with dots representing individual cases. OS, open surgery; RS, robotic surgery; and CI, confidence interval.

A drop in IFNγ after OS caused cytokine concentrations at 24 h post-incision to be higher by 1.8× following RS. Because of the opposite surgery-related trends, IL-17A and IP-10 concentrations at 72 h post-incision were significantly higher in RS—by 2.3× in the case of IL-17A and by 1.6× in the case of IP-10 (Figure 2).

#### 2.1.2. Inducers and Mediators of Th2 Response

Concentrations of all evaluated Th2 interleukins differed over time and the time-course was significantly affected by surgical approach (significant T and S × T factors). Surgery approach affected initial Th2 response with maximal effect at 8 h.

**Figure 3:**
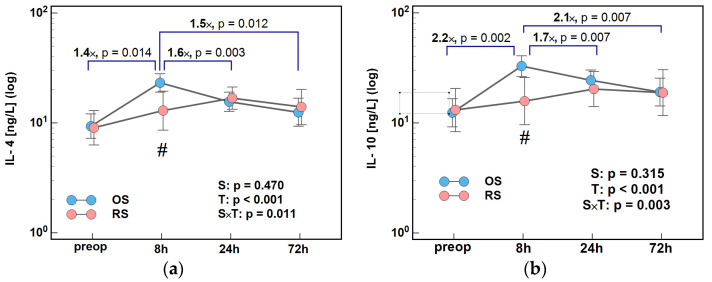
The effect of surgery type on changes in Th2 cytokine concentration during the early postoperative period: (**a**) IL-4 and (**b**) IL-10. Data were analyzed using repeated measures of analysis of variance (ANOVA) and its results are presented as probabilities (p) of effect significance for surgery (S) and time (T), and their interaction (S × T). Data are presented as geometric means with 95% CI (dots with whiskers) with significant differences in cytokine concentration at a given time-point marked by hash (#) and differences in relative change between time-points (ratios) marked by connectors with magnitude of difference. OS, open surgery; RS, robotic surgery; preop, preoperative cytokine concentration; and CI, confidence interval.

Two distinct patterns were observed. IL-4 and IL-10 peaked at 8 h following OS and then were steadily decreasing till 72 h. Following RS, they were steadily increasing till 24 h and dropped at 72 h. As a result, there were significant differences in 8/0 h, 24/8 h, and 72/8 h ratios between both surgery groups, more pronounced in the case of IL-10 (Figure 3).

**Figure 4:**
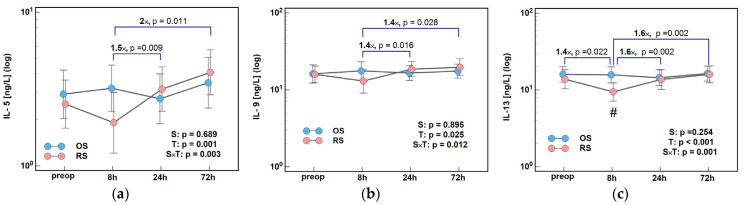
The effect of surgery type on changes in Th2 cytokine concentration during the early postoperative period: (**a**) IL-5; (**b**) IL-9; and (**c**) IL-13. Data were analyzed using repeated measures of analysis of variance (ANOVA) and the results are presented as probabilities (p) of effect significance for surgery (S) and time (T), and their interaction (S × T). Data are presented as geometric means with 95% CI (dots with whiskers) with significant differences in cytokine concentration at a given time-point marked by hash (#) and differences in relative change between time-points (ratios) marked by connectors with magnitude of difference. OS, open surgery; RS, robotic surgery; preop, preoperative cytokine concentration; and CI, confidence interval.

IL-5, IL-9, and IL-13 displayed another pattern with a toned-down elevation at 8 h and a minimum concentration at 24 h following OS. Following RS, interleukins dropped at 8 h and were subsequently increasing till 72 h. Consequently, the 24/8 h and 72/8 h ratios for IL-5, IL-9, and IL-13, and the 8/0 h ratio for IL-13, differed significantly between both surgery groups (Figure 4).

**Figure 5:**
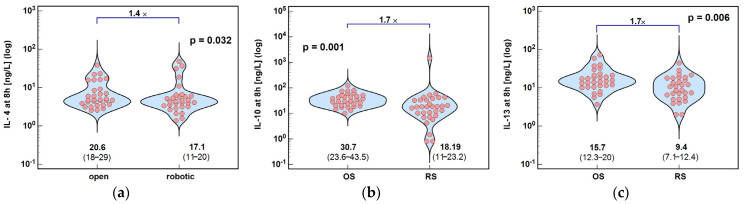
Surgery type effect on concentrations of cytokines at 8 h post-incision: (**a**) IL-4 at 8 h; (**b**) IL-10 at 8 h; and (**c**) IL-13 at 8 h. Data were analyzed using *t*-test for independent samples or Mann–Whitney *U* test. Test results are presented as means or medians, respectively, with 95% CI and probability *p*. The magnitude of between-group differences is indicated by numbers above connectors. Data distribution is illustrated by violin plots with dots representing individual cases. OS, open surgery; RS, robotic surgery; and CI, confidence interval.

The concentrations of IL-4, IL-10, and IL-13 at 8 h post-incision were significantly lower following RS than OS, by 1.4×, 1.7×, and 1.7×, respectively (Figure 5).

### 2.2. Growth Factors

#### 2.2.1. Angiogenic Growth Factors

**Figure 6:**
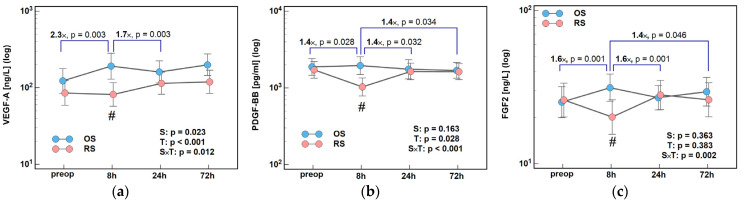
The effect of surgery type on changes in concentration of angiogenic growth factors during the early postoperative period: (**a**) VEGF-A; (**b**) PDGF-BB; and (**c**) FGF-2. Data were analyzed using repeated measures of analysis of variance (ANOVA) and its results are presented as probabilities (p) of effect significance for surgery (S) and time (T), and their interaction (S × T). Data are presented as geometric means with 95% CI (dots with whiskers) with significant differences in cytokine concentration at a given time-point marked by hash (#) and differences in relative change between time-points (ratios) marked by connectors with magnitude of difference. OS, open surgery; RS, robotic surgery; preop, preoperative cytokine concentration; and CI, confidence interval.

Concentrations of VEGF-A, PDGF-BB, and FGF2 dropped—to varying degrees—at 8 h following RS but peaked following OS. As a result, the 8/0 h and 24/8 h ratios for VEGF-A, PDGF-BB, and FGF2, and the 72/8 h ratio for PDGF-BB and FGF2 differed significantly between both surgery groups, most pronouncedly in the case of VEGF-A (Figure 6).

**Figure 7:**
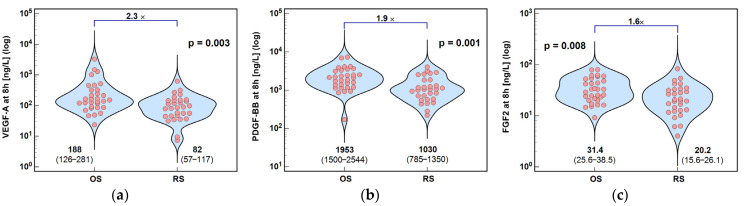
Surgery type effect on concentrations of angiogenic growth factors at 8 h post-incision: (**a**) VEGF-A; (**b**) PDGF-BB; and (**c**) FGF2. Data were analyzed using *t*-test for independent samples or Mann–Whitney *U* test. Test results are presented as means or medians, respectively, with 95% CI and probability *p*. The magnitude of between-group differences is indicated by numbers above connectors. Data distribution is illustrated by violin plots with dots representing individual cases. OS, open surgery; RS, robotic surgery; and CI, confidence interval.

Concentrations of VEGF-A, PDGF-BB, and FGF2 at 8 h post-incision were significantly lower following RS than OS by, 2.3×, 1.9×, and 1.6×, respectively (Figure 7).

#### 2.2.2. Colony-Stimulating Factors

**Figure 8:**
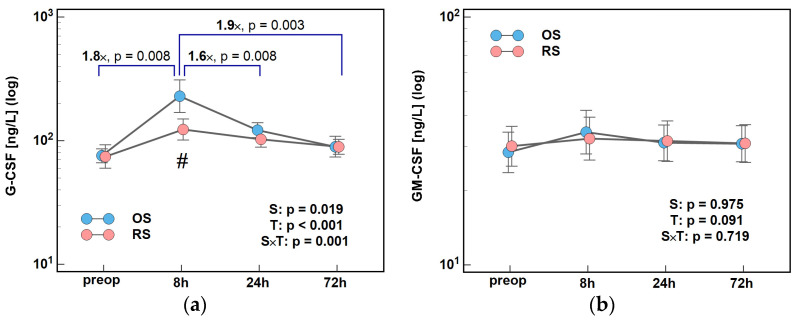
The effect of surgery type on changes in concentration of colony-stimulating factors during the early postoperative period: (**a**) G-CSF and (**b**) GM-CSF. Data were analyzed using repeated measures of analysis of variance (ANOVA) and its results are presented as probabilities (p) of effect significance for surgery (S) and time (T), and their interaction (S × T). Data are presented as geometric means with 95% CI (dots with whiskers) with significant differences in cytokine concentration at a given time-point marked by hash (#) and differences in relative change between time-points (ratios) marked by connectors with magnitude of difference. OS, open surgery; RS, robotic surgery; preop, preoperative cytokine concentration; and CI, confidence interval.

Concentrations of G-CSF differed significantly with time and between groups (significant T and S factors) with surgery type affecting the G-CSF dynamics (significant interaction factor). In both surgery groups, G-CSF peaked at 8 h post-incision and then its concentrations steadily decreased. Both the initial rise and subsequent drop were greater following OS. The 8/0 h, 24/8 h, and 72/8 h ratios were higher following OS by 1.8×, 1.6×, and 1.9×, respectively (Figure 8a). Subtle changes in GM-CSF concentrations during the early postoperative period were not altered by surgery type (Figure 8b).

**Figure 9:**
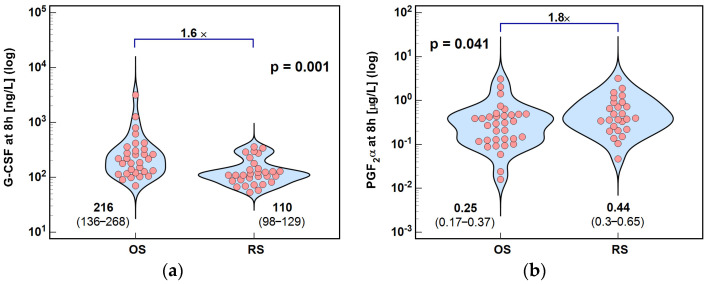
Surgery type effect on concentrations at 8 h post-incision of (**a**) G-CSF and (**b**) PGF_2_α. Data were analyzed using *t*-test for independent samples or Mann–Whitney *U* test. Test results are presented as means or medians, respectively, with 95% CI and probability *p*. The magnitude of between-group differences is indicated by numbers above connectors. Data distribution is illustrated by violin plots with dots representing individual cases. OS, open surgery; RS, robotic surgery; and CI, confidence interval.

Consistently, G-CSF concentrations at 8 h post-incision were significantly higher—by 1.6×—in OS than in RS patients (Figure 9a).

### 2.3. Lipid Mediators of Inflammation, Immunity, and Angiogenesis

**Figure 10:**
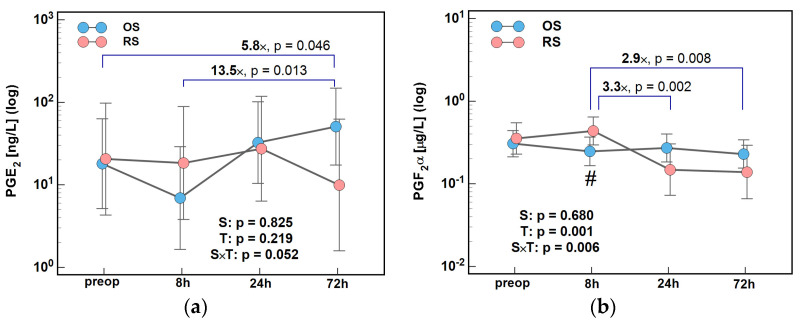
The effect of surgery type on changes in concentration of lipid mediators during the early postoperative period: (**a**) PGE_2_ and (**b**) PGF_2_α. Data were analyzed using repeated measures of analysis of variance (ANOVA) and the results are presented as probabilities (p) of effect significance for surgery (S) and time (T), and their interaction (S × T). Data are presented as geometric means with 95% CI (dots with whiskers) with significant differences in cytokine concentration at a given time-point marked by hash (#) and differences in relative change between time-points (ratios) marked by connectors with magnitude of difference. OS, open surgery; RS, robotic surgery; preop, preoperative cytokine concentration; and CI, confidence interval.

The type of surgical procedure affected changes in PGE_2_ and PGF_2_α concentrations over time, significantly so in the case of PGF_2_α. PGE_2_ dropped at 8 h and was subsequently increasing till 72 h following OS. Following RS, PGE_2_ concentrations were stable during the first 24 h but dropped afterwards. As a result, the ratios of 72/0 h and 72/8 h were higher, by 5.8× and 13.5×, respectively, in OS than in RS patients (Figure 10a). PGF_2_α concentrations initially decreased slightly following OS and increased following RS, so that at 8 h post-incision, they were significantly higher in RS patients (Figure 9b). Afterwards, PGF_2_α concentrations remained stable in the OS group but dropped at 24 h in the RS group, so that the 24/8 h and 72/8 h ratios were lower by 3.3× and 2.9×, respectively, following RS (Figure 10b).

**Figure 11:**
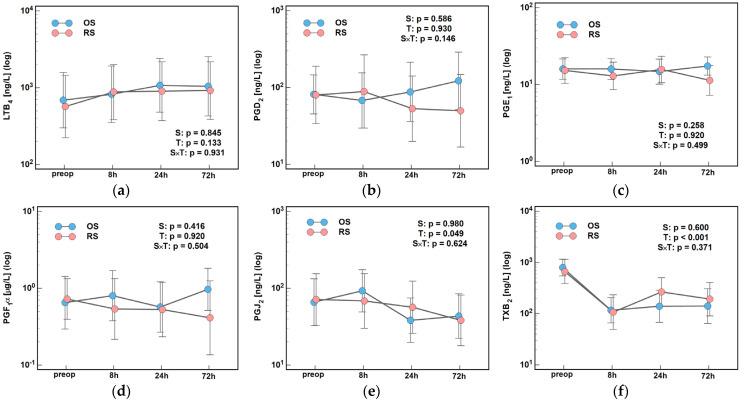
The effect of surgery type on changes in concentration of lipid mediators during the early postoperative period: (**a**) LTB_4_; (**b**) PGD_2_; (**c**) PGE_1_; (**d**) PGF_1_α; (**e**) PGJ_2_; and (**f**) TXB_2_. Data were analyzed using repeated measures of analysis of variance (ANOVA) and the results are presented as probabilities (p) of effect significance for surgery (S) and time (T), and their interaction (S × T). Data are presented as geometric means with 95% CI (dots with whiskers). OS, open surgery; RS, robotic surgery; preop, preoperative cytokine concentration; and CI, confidence interval.

Time, surgery type, or their interactions had no significant impact on remaining lipid mediators, except for PGJ_2_ and TXB_2_, the concentrations of which changed significantly over time (Figure 11).

## 3. Discussion

The perioperative period plays a critical role in shaping patient recovery and cancer outcomes after curative tumor resection. Surgical stress triggers immunosuppression within hours of surgery. It lasts for several days and diminishes cell-mediated antimicrobial and anti-tumor immunity [50]. As surgery–induced immunosuppression correlates with the extent of tissue trauma [50], the minimally invasive robot-assisted approach may help in its alleviation. Despite this, there is little research data proving the concept on a molecular basis. To the best of our knowledge, this is the first study to evaluate the stress response to surgical trauma by analyzing perioperative dynamics of key primary messengers—including cytokines, growth factors, and prostanoids—and to compare the effects of surgical approach (robotic vs. open surgery).

We found the most pronounced surgery-related changes in the concentrations of these mediators to occur within the first 24 h post-incision. Of crucial relevance is the observation that they involved components of both SIR/Th1 and CARS/Th2. The simultaneous activation of these opposing pathways constitutes a mixed antagonist response syndrome (MARS) [51]. MARS has been associated with higher mortality than SIRS and CARS [52]. It may evolve into persistent low-grade inflammation accompanied by profound immunosuppression, leading to catabolic states and gradual loss of lean body mass [53]. This condition, known as “persistent inflammation, immunosuppression, and catabolism syndrome” (PICS) is characterized by immune paralysis and represents the most severe manifestation of chronic critical illness (CCI). PICS is believed to contribute to rehabilitation failure, repeated hospitalizations, delayed multi-organ failure, and, ultimately, indolent death [51,52,53,54,55]. Although PICS itself is not oncogenic, it fosters a tumor-promoting environment and impairs host defenses against cancer and pathogens. It increases the susceptibility of cancer patients to infections, malnutrition, and toxicity, reducing their tolerance to anticancer therapies [51,54,55,56]. With an aging population and improvements in acute care, the incidence of CCI and PICS are rising, even as associated mortality declines [35,57]. This trend underscores the urgent need for preventive strategies, including identifying new pharmacological targets [35,53,54]. Understanding the interplay among primary messengers—which orchestrate the immune response to surgical trauma—is therefore of critical importance.

Previous studies have shown that RS attenuates the activation of the hypothalamic–pituitary–adrenal (HPA) axis [45], which governs immune responses [58] and lessens both SIR and CAR [46,47]. It has been evidenced by diminished cortisol elevation [45] and reduced postoperative peaks in pro-inflammatory IL-6, acute-phase reactants (C-reactive protein and procalcitonin) [46], chemokines IL-8 and MCP-1 [47], and the anti-inflammatory IL-1 receptor antagonist (IL-1ra) [46]. Additionally, RS appeared to mitigate postoperative lymphocytopenia, potentially via IL-7 activity [44], and favored a Th1-dominant response while preserving immunocompetence, as indicated by upregulation of HLA-DR expression on postoperative day three [43]. The present study reinforces these findings: lower postoperative IL-6 elevation—a known Th1 suppressor [59]—was paralleled in RS by increased IFNγ, a hallmark Th1 cytokine. Moreover, other Th1-associated cytokines, such as IL-12(p70) and IP-10, rose steadily between 8 and 72 h post-incision. These observations support the notion of preserved cellular immunity after RS [42,60,61]. IFNγ, primarily produced by NK and NKT cells (with minor contributions from Th1 and CD8+ T cells) [59], is suppressed by elevated cortisol, acute-phase proteins, and immunosuppressive cytokines like IL-10 and TGFβ during surgical stress [42,59,62]. The steady increase in IFNγ observed after RS likely reflects a blunted cortisol [45] and acute-phase [46] responses. Together with reduced IL-10 elevation seen in the current study, they imply a better preservation of NK-cell function and anti-tumor immunity. Furthermore, IP-10 levels declined after OS but consistently rose after RS. As an IFNγ-inducible cytokine, IP-10 enhances NK and T-cell recruitment and cytotoxicity while promoting Th1 polarization [63,64,65]. IL-12(p70), produced by dendritic cells and macrophages, enhances both innate and adaptive immunity by stimulating T- and NK-cell cytotoxicity and IFNγ production [59,62]. Together, IFNγ and IL-12(p70) promote nitric oxide synthase expression in macrophages, a hallmark of M1 anti-tumor polarization [62], and suppress TGFβ signaling by downregulating its receptor TGFβRII [66]. Muted elevation or even transient decreases in pro-inflammatory IFNγ and IL-12(p70) during early RS response reflects an attenuated SIR, associated with reduced oxidative stress [48,67], improved tissue preservation, and lower risk of multi-organ failure [36,54]. Taken together, these findings indicate that RS may attenuate surgery-induced immunosuppression by preserving Th1-polarized cellular responses and NK-cell function. The maintenance of IFNγ, IL-12(p70), and IP-10 activity suggests sustained cytotoxic and anti-tumor immunity, underscoring the potential of RS to better preserve perioperative immune competence compared with conventional surgical approaches.

The modest rise in IL-17A levels after RS—contrasted with its steady decline following OS—may enhance resistance to postoperative infections, consistent with lower infection rates observed after RS [12,24]. IL-17A, primarily secreted by Th17, CD8+, and γδ T cells, supports protective antibacterial and antifungal immunity by promoting antimicrobial peptide production and epithelial barrier integrity [68,69]. Specifically, it acts on fibroblasts and endothelial and epithelial cells, prompting them to release modulatory cytokines in order to promote immune cells proliferation and maturation. In acute situations, such as surgical injury, IL-17A provides protection by upregulating the expression of defensins, COX-2, TNFα, G-CSF, and GRO1 [70]. It also enhances wound healing and bolsters Th1 immunity. IL-17A induces expression of matrix metalloproteinases and stimulates angiogenesis and extracellular matrix remodeling. Importantly, it enables mucosal healing by triggering proliferation of gut epithelial cells and restoring tight junctions [70,71]. While IL-17A plays dual roles in cancer biology [71,72,73], emerging evidence suggests that its anti-tumor or pro-tumor effects depend on its cellular source. Intraepithelial IL-17A may enhance anti-tumor responses via recruitment of cytotoxic immune cells [72]. Although IL-17 alone has weak angiogenic activity, its proangiogenic effects become more pronounced in the presence of factors like FGF2 [68]. In our study, concentrations of FGF2, VEGF-A, and PDGF-BB remained unchanged postoperatively, while proangiogenic prostaglandins (PGE_2_ and PGF_2_α) declined. These observations align with earlier findings on RS moderating a perioperative elevation of IL-8 and MCP-1 [47], chemokines which are potent inductors of angiogenesis [74,75,76]. FGF2, VEGF-A, and PDGF-BB are key players in both sprouting and intussusceptive neoangiogenesis, and VEGFA/VEGFRs signaling and MCP-1 are also involved in vasculogenesis by engaging endothelial progenitor cells [77,78]. While angiogenesis is necessary for post-trauma recovery, including anastomosis and wound healing [79,80], excessive secretion of its mediators may facilitate growth of residual cancer cells and promote metastasis [81]. Collectively, our observations demonstrate the modest IL-17A increase after RS, which may enhance antimicrobial defense and wound healing, while stable proangiogenic factor levels suggest restrained angiogenic activity, potentially supporting recovery without promoting tumor growth.

RS was also associated with dampened Th2 responses, demonstrated by lower early postoperative levels of key immunosuppressive interleukins IL-10 and IL-4 as well as decreased IL-5, IL-9, and IL-13. IL-4, IL-5, and IL-13 are overexpressed in colorectal cancer (CRC) [38,82,83]. IL-4 is a prototypical mediator of Th2 immunity, which exerts its anti-inflammatory function in the bowel by stimulating glucocorticoid synthesis by intestinal cells [84] and hampering T-cell activation. It also promotes macrophage polarization into the anti-inflammatory and immunosuppressive M2 phenotype with further stimulation of their activity [85,86]. IL-4 facilitates cancer-cell migration [83], epithelial–mesenchymal transition [82], and immune evasion [84], and its blocking has been shown to improve the effectiveness of cancer immunotherapy [87]. Given the role of IL-4, its attenuation following RS indicates a more favorable perioperative immune profile, potentially limiting cancer-cell survival and spread.

Depending on the context, IL-10 may exert both tumor-promoting and -suppressing effects. However, its knockout has appeared to be beneficial as it sensitized cancer cells to DC-based immunotherapy and restored anti-tumor Th1 immunity while reducing immunosuppressive Tregs and myeloid-derived suppressor cells (MDSCs) [85]. IL-10 hinders anti-tumor responses of NK cells [62] but it also suppresses the release of Th1 mediators from eosinophils [88]. Li et al. [89] reported tumor excision to downregulate serum IL-10. It was lower on the seventh day and declined further till the discharge day, which agrees well with the downward trend observed at the end of our short follow-up. High IL-10 concentrations were predictive of cancer recurrence [89]. Therefore, its twice-lower postoperative increase after RS may suggest reduced immunosuppression and can therefore be considered beneficial in terms of antimicrobial and anti-tumor defenses.

IL-5 supports adaptive humoral immunity by promoting B-cells proliferation and differentiation, facilitating their survival, and enabling functionality [90]. It is also a key growth, survival, and differentiation-inducing factor for eosinophils of the innate arm of immunity [88,90]. IL-5, together with IL-13, sensitizes eosinophils to their chemoattractant, eotaxin, guiding their homing to the bowel. There, eosinophils are involved in inflammatory responses to parasites, mucosal healing, and cancer-cell surveillance. Depending on the context and cytokine milieu, eosinophils may contribute to either Th1 or Th2 immunity and switch their secretome profile between these two to either support or hinder tumor growth [88]. Therefore, the rebound of IL-5 levels after an initial dip may support humoral immunity and eosinophil-mediated tumor surveillance—an established favorable prognostic factor in CRC [91]. IL-9 and IL-13 followed similar trends, consistent with a moderated Th2 response.

We also found that RS resulted in a less pronounced increase in G-CSF, a growth factor involved in neutrophil proliferation and mobilization [92]. Excessive G-CSF can drive tissue damage through overactive neutrophils and foster immunosuppression by inducing IL-10, inhibiting IL-12, and promoting Treg and Th2 responses [92]. PICS is characterized by aberrant myelopoiesis and deviant MDSCs mediate some of G-CSF’s effects, including impairing T-cell expansion and survival by arginine depletion [53,57]. Our findings of reduced G-CSF align with previous observations of preserved arginine levels after RS [48]. Moreover, G-CSF is implicated in tumor progression via the promotion of proliferation and neovascularization [92], and impaired NK-cell cytotoxicity has been linked to increased metastases in animal models [61]. Thus, the subdued G-CSF response seen after RS may help limit postoperative immunosuppression, a key contributor to surgery complications such as infections, sepsis, and delayed wound healing, as well as to the hampered clearing of residual cancer cells.

Finally, prostaglandins—key lipid mediators released in response to surgical trauma—can impair postoperative immune surveillance by reducing NK-cell activity, thereby providing growth advantage to residual cancer cells [93]. In our study, RS was associated with a significant decline in PGE_2_, the principal COX-2 product, whereas OS led to a sustained increase with differences reaching up to 13.5-fold. PGE_2_ plays a pivotal role in CRC progression by promoting tumor proliferation, survival, and dissemination [94,95]. It also facilitates the re-population of cancer stem cells, enabling resistance to therapy and tumor recurrence after surgery [96]. Experimental inhibition of the COX-2/PGE_2_ axis has been shown to improve survival after primary tumor resection by reducing metastasis formation [97], underscoring its clinical significance in postoperative cancer control. PGE_2_ has also been shown to upregulate checkpoint receptor PD1 expression in CD8+ lymphocytes and macrophages, hindering proliferation and cytotoxicity of T cells and reducing phagocytic potential of macrophages [98]. Moreover, it might interfere with NK and DC recruitment, hamper their proliferation and maturation, and reduce cytokine secretion and cytotoxicity of NK cells [96]. Furthermore, PGE_2_ has been reported to skew DC and macrophage polarization into cancer-tolerogenic phenotypes [99].

Similarly, PGF_2_α, another COX-2-derived mediator overexpressed in CRC [100], showed a more pronounced decline following RS than OS. Mechanistically, PGF_2_α enhances motility and invasiveness of colorectal cancer cells [101] and enables their resistance to oxaliplatin therapy by mitigating ROS production, thus protecting tumor DNA from oxidative damage [100]. Given their pro-tumorigenic properties, the attenuation of both COX-2/PGE_2_ and COX-2/PGF_2_α responses after RS may translate into clinically meaningful improvements in postoperative immune function and long-term cancer outcomes.

In summary, RS induces attenuated SIR and CAR responses, reducing the risk of immune dysregulation syndromes and facilitating earlier restoration of homeostasis. The attenuated catabolic response, indicated by reduced cortisol dynamics [45], further supports improved postoperative recovery and reduces risk of CCI and PICS. The favorable immune profile observed after RS—including better balanced Th1/Th2 immunity and reduced immunosuppression—may explain better clinical outcomes in our cohort, such as less leukopenia and neutrophilia [44], fewer infections, shorter hospital stays, and faster bowel function recovery [12]. In oncologic patients, preserved anti-tumor immunity post-RS may help to reduce the risk of adverse oncological outcomes [102].

However, RS typically involves longer operative times, which may increase surgical stress and anesthetic exposure [103], potentially obscuring its immunological advantages. All patients in our study received a uniform anesthetic regimen: induction with propofol followed by maintenance with sevoflurane. While propofol exerts anti-inflammatory and anti-tumor effects, sevoflurane is known to promote immunosuppression and tumor angiogenesis (via HIF-1α), and to impair NK- and T-cell function through oxidative stress pathways [103]. Therefore, minimizing surgical time and avoiding volatile anesthetics may further enhance the immunological benefits of RS in colorectal cancer surgery.

## 4. Limitations

This is the first study to analyze fluctuations in the concentrations of growth factors, selected interleukins, and lipid inflammatory mediators in the early perioperative period. It is also one of the few studies attempting to compare the body’s response to surgical stress depending on the technique used—robot-assisted or open surgery—which may translate into more favorable clinical outcomes following robot-assisted procedures. However, due to the pioneering nature of robot-assisted CRC surgery in our country at the time of the project, the interpretation of the study results should take into account the following limitations.

The first limitation is the lack of randomization, as the final decision to undergo robotic surgery was made by the patient. Despite this, both patient groups were well matched in terms of demographic and clinical characteristics. The second limitation, resulting from the high cost of robotic surgery, is the relatively small study population. Therefore, our observations should be confirmed in larger studies. Another limitation, related to the pioneering nature of robotic procedures, is the greater experience of the surgical team with open surgeries compared to robotic ones. Nevertheless, this difference in experience would have favored open surgery and may have potentially led to an underestimation of the benefits of the robotic approach.

## 5. Materials and Methods

### 5.1. Patients

#### 5.1.1. Study Type and Design

Blood samples during a short follow-up (up to 72 h) of 61 patients with CRC undergoing OS (*n* = 31) or RS (*n* = 30) were collected during realization of a prospective nonrandomized study “Comparison of inflammatory, immune and angiogenic response as well as homeostasis in colorectal cancer patients undergoing robot-assisted and classic open surgery”. This study constituted a part of the “Wrovasc—Integrated Cardiovascular Centre” project realized by Regional Hospital in Wroclaw, Poland, in the years 2012–2015.

Patients consecutively admitted to the Department of Surgical Oncology for curative resection of colorectal tumors were included in the study if they were ≥18 years old, had confirmed diagnosis of colorectal cancer and had not been treated yet, and if they consented to the study. They were excluded if an informed consent was withdrawn/not given, an emergency surgery or en bloc multi-visceral resection was required, were classified as ASA > 3, had gross metastatic disease or tumors which were not amenable to resection due to local advancement, had synchronous malignancies or severe mental or systemic diseases including diabetes, cardiovascular and/or respiratory distress, or immunological conditions requiring systemic corticosteroids. For the current study, only patients for whom blood samples were available from all time-points were included.

#### 5.1.2. Patients’ Characteristics

Patients underwent standard preoperative workups consisting of colonoscopy and computed tomography of the abdomen. Patients with rectal cancers underwent computed tomography and magnetic resonance imaging of the pelvis. Their general health condition was evaluated using Physical Status Classification System of the American Society of Anesthesiologists (ASA). The UICC staging system tumor-node-metastasis (TNM7th) was applied to determine cancer advancement.

Data on patients’ demographics and health conditions, laboratory parameters (including total blood count), and cancer advancement were collected prospectively. Based on neutrophil and lymphocyte counts, the neutrophil-to-lymphocyte (NLR) index was calculated prior to surgery and on postoperative day one to assess immune system mobilization.

Patients with CRC undergoing OS or RS were well matched regarding their demographics, general health condition, cancer advancement, and surgery extension and severity, except for longer operation time in RS. Their baseline characteristics, described in detail in our prior research [46,47,49], are summarized in Table 1.

#### 5.1.3. Treatment

The final decision regarding the type of procedure rested with the patient, advised by the attending surgeon, who took into account the patient’s individual circumstances. RS was performed by two surgeons with credentials in robotic surgery using the Da Vinci Si system (Intuitive Surgical^®^, Sunnyvale, CA, USA). Standard care protocols and preventive measures were introduced and included mechanical bowel preparation and administration of antibiotics and anticoagulants (LMWH). Standardized general anesthesia with propofol, rocuronium, and fentanyl (*i.v.*) was applied for induction and maintained with sevoflurane. No epidural or local anesthesia was used. Before or directly after waking up, patients were given metamisol. Parenteral opioids were administered for postoperative pain control. Routinely used surgical drains were removed on the 1st/2nd postoperative day.

### 5.2. Ethical Considerations

The study protocol was approved by the Medical Ethics Committees of Regional Specialist Hospital (#KB/nr 1/rok 2012 from 26 June 2012) and Wroclaw Medical University (#KB 660/2024 from 22 November 2024). The study was conducted in accordance with the principles of the Good Clinical Practice and the Helsinki Declaration of 1975, as revised in 1983, and informed consent has been obtained from all patients.

### 5.3. Preparative and Analytical Methods

#### 5.3.1. Blood

Blood was sampled prior to any medical procedure and at three time-points during the early postoperative period, that is, at 8, 24, and 72 h post-incision. It was drawn by venipuncture into serum separator tubes. Blood was let to clot for 30 min at room temperature and subsequently centrifuged for 15 min at 720× *g* and room temperature. Collected sera were stored in aliquots at -80° till examination.

#### 5.3.2. Profiling Cytokines

Luminex xMAP^®^ technology was used to profile circulating growth factors: VEGF-A, PDGF-BB, FGF2, G-CSF, and GM-CSF; interleukins: IL-2, IL-4, IL-5, IL-9, IL-10, IL-13, IL-15, and IL-17A; and cytokines: IFNγ and IFNγ-induced protein 10 (IP-10). It is based on flow cytometry and uses antibody-conjugated magnetic microspheres, allowing for simultaneous quantification of multiple targets by fluorescence reading conducted in real time. The BioPlex 200 platform with HRF (BioRad, Hercules, CA, USA) was used for measurements, which were conducted in two technical replicates. A complete set of samples from a given patient, collected at different time-points, was assessed within the same run. The abovementioned analytes were selected from commercially available human cytokine panels (BioRad). The bioassays were conducted according to the manufacturer’s instructions. Standard curves were drawn using 4- or 5-PL logistic regression and the data were analyzed using BioPlex Manager 6.0 software (BioRad). In a number of cases the concentrations of IL-2, IL-15, and IL-17A were below the detection limit of the assay and IL-2 and IL-15 interleukins had to be excluded from further analysis, while evaluation of IL-17A was conducted on 35 patients (17 after RS and 18 after OS).

#### 5.3.3. Profiling Lipid Inflammatory Mediators

Mass spectrometry was used to profile prostaglandins (and/or their stable metabolites) 13,14-dihydro-PGE_1_, PGE_2_, 6-ketoPGF_1_α (PGI_2_ metabolite), PGF_2_α, PGD_2_, 15-deoxy- Δ12,14- PGJ_2_ (PGD_2_ metabolite), thromboxane TXB_2_ (TXA_2_ metabolite), and leukotriene LTB_4_ (LTA_4_ metabolite). For clarity, 13,14-dihydro-PGE_1_ is further referred to as PGE_1_ and 15-deoxy-12,14- PGJ_2_ as PGJ_2_.

##### Materials

Standards of TXB_2_, LTB_4_, PGD_2_, PGE_2_, 6-ketoPGF_1_α, PGF_2_α, 15-deoxy- Δ12,14- PGJ_2_, and 13,14-dihydro-PGE_1_ and their isotope-labeled standards were procured from Cayman Chemical Company (Ann Arbor, MI, USA). Methanol, acetonitrile (ACN), ethyl acetate, water, and formic acid (FA) were acquired from Witko (Warsaw, Poland).

##### Targeted Metabolomic Analysis

Samples were subjected to a quantitative analysis. Compounds were separated using a triple quadrupole mass spectrometer Xevo Absolute from Waters Corp. (Milford, MA, USA). Separation of eicosanoids was achieved based on the previously described method [104]. Briefly, 100 µL of samples or calibration standards, placed in 2 mL Eppendorf tubes, were mixed with 20 µL of 0.2% FA and 10 µL of internal standards in methanol for 1 min at 1100 RPM and 25 °C. Afterwards, 200 µL of ACN and 250 µL of ethyl acetate were added and mixed for 10 min at 1100 RPM and 25 °C. These mixtures were subsequently centrifuged at 4 °C for 7 min at 15,000 RCF. Supernatant aliquots of 370 µL were evaporated to dryness and re-dissolved in 25 µL of 20% ACN in water before analysis.

Chromatographic separation of metabolites was conducted on BEH Shield C18 column (100 mm × 2.1 mm i.d., 1.7 µm; Waters). Data acquisition for all compounds was carried out on MassLynx Software 10.50 (Waters) in multiple-reaction-monitoring mode (MRM).

### 5.4. Statistical Analysis

Raw data from technical replicates were averaged and submitted to statistical analysis following logarithmic transformation, used to normalize data distribution and improve homogeneity of variances, and were tested with the D’Agostino–Pearson test for normality and Levene’s test, respectively. Potential effect of surgical approach on a time-course of studied cytokines and lipids was evaluated by means of repeated measures of analysis of variance (ANOVA) with time as a “within factor” and OS/RS as a “between factor” (group affiliation). Low *p* value (≤0.05) accompanying F statistics for a between effect (denoted as “S”) indicates significant differences between open and robotic groups whilst low *p* value for within effect (denoted as “T”) indicates significant differences between measurements. Low *p* value for an interaction factor (herein time and surgery interaction, denoted as S × T) indicates significant impact of surgery group affiliation on differences between measurements.

To assess the dynamics of observed changes in cytokine and lipid concentrations and to quantify their effect, ratios between concentrations at given time-points (e.g., concentration at 8 h in relation to preoperative measurement (8/0 h), at 24 h in relation to 8 h (24/8 h), at 72 h in relation to 8 h (72/8 h), etc.) were calculated and compared between open and robotic surgery groups. Depending on the normality of distribution and homogeneity of variances, these calculated ratios or cytokine concentrations at given time-points were compared using *t*-test for independent samples (normal distribution, homogeneous variances) with Welch correction (normal distribution, non-homogeneous variances) and presented as means, or with Mann–Whitney *U* test (non-normal distribution) and presented as medians with 95% confidence interval (CI) around them.

Other tests, such as one-way ANOVA, Kruskal–Wallis H test, and the frequency distribution tests Fisher exact test (2 × 2 tables) and χ^2^ test (2 × 3 tables or higher), were applied to compare demographics, histological data, and laboratory parameters as well as surgery-related data between OS and RS groups.

All applied tests were two-sided, and probability was set at ≤0.05. All statistical analyses were conducted using MedCalc^®^ Statistical Software version 23.0.2 (MedCalc Software Ltd., Ostend, Belgium; https://www.medcalc.org; 2024) licensed to Małgorzata Krzystek-Korpacka.

## 6. Conclusions

Robot-assisted surgery (RS) for colorectal cancer was associated with a more favorable postoperative immune profile compared to open surgery. Patients undergoing RS showed reduced SIR and muted coexisting CAR, preserved anti-tumor Th1 with muted immunosuppressive Th2 immunity, and lower levels of proangiogenic factors. These immune-modulating effects may help limit postoperative complications, support faster recovery, and reduce the risk of CCI and PICS. By maintaining postoperative immune competence, RS may help to mitigate recurrence-promoting mechanisms and ultimately improve long-term oncologic outcomes in patients undergoing curative colorectal cancer resection, despite longer operative times. Further validation in larger cohorts with a follow-up allowing for assessment of long-term outcomes is warranted.

## Figures and Tables

**Table 1:** Characteristics of patients, disease, and surgery.

Characteristics	Open Surgery (OS)	Robotic Surgery (RS)	*p*
*N*	31	30	-
Sex (F/M), *n*	14/17	7/23	0.127 ^1^
Age [yrs.]	68 (65–76)	67 (62–72)	0.302 ^2^
BMI [kg/m^2^]	26 (25–28)	25 (24–28)	0.716 ^2^
ASA (1/2/3), *n*	6/20/5	5/20/5	0.830 ^3^
NLR	3.17 (2.59–3.49)	3.58 (2.81–4.24)	0.194 ^2^
pTNM (0/1/2/3/4)	2/2/15/9/3	2/3/11/12/2	0.839 ^3^
T (tis/1/2/3/4)	2/1/1/20/7	2/0/5/16/7	0.393 ^3^
N (0/1/2)	19/4/8	16/8/6	0.395 ^3^
M (Y/N)	28/3	28/2	0.970 ^1^
G (x/1/2/3/4)	1/4/21/4/1	2/6/17/5/0	0.601 ^3^
Tumor (LC/RE/RC)	11/14/6	7/11/12	0.199 ^3^
LoS [min]	125 (115–150)	205 (191–240)	<0.001 ^2^
HLN, *n*	14 (12–17)	13.5 (12–17)	0.700 ^2^
Transfusion (Y/N), *n*	26/5	28/2	0.425 ^1^

*N*, number of observations; p, probability; F, females; M, males; yrs., years; BMI, body mass index; ASA, American Society of Anesthesiologists Physical Status Classification System; NLR, neutrophil-to-lymphocyte ratio; pTNM, pathological cancer stage (tumor-node-metastasis); T, local advancement; N, lymph node involvement; M, distant metastasis; tis, tumor in situ; Y/N, yes/no; G, histological grade; LC, left colon; RE, rectum; RC, right colon; LoS, length of surgery; min, minutes; HLN, harvested lymph nodes; and CI, confidence interval. ^1^ Fisher exact test; ^2^ data analyzed with Mann–Whitney U test and presented as medians (95% CI); and ^3^ chi-squared test.

## Data Availability

Data are available from the corresponding authors upon reasonable request.

## Abbreviations

The following abbreviations are used in this manuscript:

ACN	Acetonitrile
ASA	Physical status classification system of the American Society of Anesthesiologists
BMI	Body mass index
CAR	Compensatory anti-inflammatory response
CARS	CAR syndrome
CCI	Chronic critical illness
CI	Confidence interval
COX-2	Cyclooxygenase 2
CRC	Colorectal cancer
F	Females
FA	Formic acid
FGF2	Basic fibroblast growth factor
G-CSF	Granulocyte colony-stimulating factor
GM-CSF	Granulocyte–macrophage colony-stimulating factor
HIF-1α	Hypoxia-inducible factor 1α
HLN	Harvested lymph nodes
IFNγ	Interferon γ
IL	Interleukin
IP-10	Interferon-gamma-inducible protein 10 kD; CXCL10
LC	Left colon
LMWH	Low-molecular-weight heparin
LoS	Length of surgery
LS	Conventional laparoscopy (laparoscopic surgery)
M	Males
MARS	Mixed antagonist response syndrome
MCP1	Monocyte chemoattractant protein-1; CCL2
MDSCs	Myeloid-derived suppressor cells
NK	Natural killer cells
NKT	Natural killer T cells
NLR	Neutrophil-to-lymphocyte ratio
OS	Open surgery
PDGF-BB	Platelet-derived growth factor BB
PG	Prostaglandin
PICS	“Persistent inflammation, immunosuppression, and catabolism syndrome”
RC	Right colon
RE	Rectum
RS	Robot-assisted surgery
SIR	Systemic inflammatory response
SIRS	SIR syndrome
TGFβ	Transforming Growth Factor β
TGFβRII	TGFβ receptor II
TNM	“Tumor-node-metastasis” cancer staging system
TX	Thromboxane
VEGF-A	Vascular endothelial growth factor A

## References

[1] (2024). American Cancer Society: Cancer Facts and Figures 2024. https://www.cancer.gov/types/colorectal.

[2] Global Cancer Observatory (GCO). https://gco.iarc.who.int/en.

[3] Zhou J., Yang Q., Zhao S., Sun L., Li R., Wang J., Wang L., Wang D. (2025). Evolving landscape of colorectal cancer: Global and regional burden, risk factor dynamics, and future scenarios (the Global Burden of Disease 1990–2050). Ageing Res. Rev..

[4] Zhang J., Ou D., Xie A., Chen D., Li X. (2024). Global burden and cross-country health inequalities of early-onset colorectal cancer and its risk factors from 1990 to 2021 and its projection until 2036. BMC Public Health.

[5] Siegel R.L., Wagle N.S., Cercek A., Smith R.A., Jemal A. (2023). Colorectal cancer statistics, 2023. CA Cancer J. Clin..

[6] Santucci C., Mignozzi S., Malvezzi M., Boffetta P., Collatuzzo G., Levi F., La Vecchia C., Negri E. (2024). European cancer mortality predictions for the year 2024 with focus on colorectal cancer. Ann. Oncol..

[7] Didkowska J.A., Barańska K., Miklewska M.J., Wojciechowska U. (2024). Cancer incidence and mortality in Poland in 2023. Nowotw. J. Oncol..

[8] Luo S., Gong J., Zhu Y., Wang L., Zhang K. (2025). Global, regional, and national burden of colorectal cancer in the elderly (aged  >  60 years): A comprehensive analysis across 204 countries and territories (1990–2021). BMC Gastroenterol..

[9] Li X., Xiao X., Wu Z., Li A., Wang W., Lin R. (2025). Global, regional, and national burden of early-onset colorectal cancer and projection to 2050: An analysis based on the Global Burden of Disease Study 2021. Public Health.

[10] Sung H., Siegel R.L., Laversanne M., Jiang C., Morgan E., Zahwe M., Cao Y., Bray F., Jemal A. (2025). Colorectal cancer incidence trends in younger versus older adults: An analysis of population-based cancer registry data. Lancet Oncol..

[11] Chakedis J., Schmidt C.R. (2018). Surgical Treatment of Metastatic Colorectal Cancer. Surg. Oncol. Clin. N. Am..

[12] Zawadzki M., Krzystek-Korpacka M., Rząca M., Czarnecki R., Obuszko Z., Witkiewicz W. (2017). Introduction of robotic surgery into a community hospital setting: A prospective comparison of robotic and open colorectal resection for cancer. Dig. Surg..

[13] Lu C.C., Lu C.T., Chang K.Y., Chun-Li W., Wu C.Y. (2024). Robot-assisted vs. laparoscopic right hemicolectomy in octogenarians and nonagenarians: An analysis of the US nationwide inpatient sample 2005–2018. Aging Clin. Exp. Res..

[14] Hettiarachchi T.S., Askari A., Rudge E., Hao L.T., Sarwar S., Dowsett D., El Hadi A., Shaikh I. (2023). Comparison of robotic vs laparoscopic left-sided colorectal cancer resections. J. Robot. Surg..

[15] Dohrn N., Klein M.F., Gögenur I. (2021). Robotic versus laparoscopic right colectomy for colon cancer: A nationwide cohort study. Int. J. Color. Dis..

[16] Baik S.H., Kwon H.Y., Kim J.S., Hur H., Sohn S.K., Cho C.H., Kim H. (2009). Robotic versus laparoscopic low anterior resection of rectal cancer: Short-term outcome of a prospective comparative study. Ann. Surg. Oncol..

[17] Chen Y.T., Huang C.W., Ma C.J., Tsai H.L., Yeh Y.S., Su W.C., Chai C.Y., Wang J.Y. (2020). An observational study of patho-oncological outcomes of various surgical methods in total mesorectal excision for rectal cancer: A single center analysis. BMC Surg..

[18] Sterk M.F.M., Crolla R.M.P.H., Verseveld M., Dekker J.W.T., van der Schelling G.P., Verhoef C., Olthof P.B. (2023). Uptake of robot-assisted colon cancer surgery in the Netherlands. Surg. Endosc..

[19] Butnari V., Sultana M., Mansuri A., Rao C., Kaul S., Boulton R., Huang J., Rajendran N. (2024). Comparison of early surgical outcomes of robotic and laparoscopic colorectal cancer resection reported by a busy district general hospital in England. Sci. Rep..

[20] Lin C.-Y., Liu Y.-C., Chen C.-C., Chen M.-C., Chiu T.-Y., Huang Y.-L., Chiang S.-W., Lin C.-L., Chen Y.-J., Lin C.-Y. (2025). Robotic-Assisted Colon Cancer Surgery: Faster Recovery and Less Pain Compared to Laparoscopy in a Retrospective Propensity-Matched Study. Cancers.

[21] Meyer J., Meyer E., Meurette G., Liot E., Toso C., Ris F. (2024). Robotic versus laparoscopic right hemicolectomy: A systematic review of the evidence. J. Robot. Surg..

[22] Zhang G., Pan S., Yang S., Wei J., Rong J., Wu D. (2024). Impact of robotic surgery on postoperative gastrointestinal dysfunction following minimally invasive colorectal surgery: Incidence, risk factors, and short-term outcomes. Int. J. Color. Dis..

[23] Law W.L., Foo D.C.C. (2017). Comparison of short-term and oncologic outcomes of robotic and laparoscopic resection for mid- and distal rectal cancer. Surg. Endosc..

[24] Laks S., Goldenshluger M., Lebedeyev A., Anderson Y., Gruper O., Segev L. (2025). Robotic Rectal Cancer Surgery: Perioperative and Long-Term Oncological Outcomes of a Single-Center Analysis Compared with Laparoscopic and Open Approach. Cancers.

[25] Ferrari D., Violante T., Novelli M., Sassun R., Sileo A., Larson D.W. (2025). Robotic Surgery in Emergency Colorectal Procedures: Analysis of Outcomes and Future Trends. J. Am. Coll. Surg..

[26] Safiejko K., Tarkowski R., Koselak M., Juchimiuk M., Tarasik A., Pruc M., Smereka J., Szarpak L. (2022). Robotic-Assisted vs. Standard Laparoscopic Surgery for Rectal Cancer Resection: A Systematic Review and Meta-Analysis of 19,731 Patients. Cancers.

[27] Ng K.T., Tsia A.K.V., Chong V.Y.L. (2019). Robotic versus conventional laparoscopic surgery for colorectal cancer: A systematic review and meta-analysis with trial sequential analysis. World J. Surg..

[28] Ravendran K., Abiola E., Balagumar K., Raja A.Z., Flaih M., Vaja S.P., Muhidin A.O., Madouros N. (2023). A Review of robotic surgery in colorectal surgery. Cureus.

[29] Simon E.F., Westfall K.M., Erozkan K., Henke L., Costedio M., Teetor T., Selfridge J.E., Steinhagen E., Charles R. (2025). The rise of robotics: Surgical approaches for rectal cancer over time. Surg. Endosc..

[30] Wang W., Liu J., Wang J., Huang J., Wang J. (2025). Comparing robot-assisted vs. laparoscopic proctectomy for rectal cancer surgical and oncological outcomes. Front. Surg..

[31] Hopkins M.B., Hawkins A.T., Tiwari V., Soda M., Martin B.J., Muldoon R.L., Ford M.M., Beck D., Geiger T.M. (2022). Is newer always better? Comparing cost and short-term outcomes between laparoscopic and robotic right hemicolectomy. Surg. Endosc..

[32] Wei P.L., Huang Y.J., Wang W., Huang Y.M. (2023). Comparison of robotic reduced-port and laparoscopic approaches for left-sided colorectal cancer surgery. Asian J. Surg..

[33] Dobson G.P. (2015). Addressing the Global Burden of Trauma in Major Surgery. Front. Surg..

[34] Bain C.R., Myles P.S., Corcoran T., Dieleman J.M. (2023). Postoperative systemic inflammatory dysregulation and corticosteroids: A narrative review. Anaesthesia.

[35] Rosenthal M.D., Moore F.A. (2016). Persistent Inflammation, Immunosuppression, and Catabolism: Evolution of Multiple Organ Dysfunction. Surg. Infect..

[36] Gentile L.F., Cuenca A.G., Efron P.A., Ang D., Bihorac A., McKinley B.A., Moldawer L.L., Moore F.A. (2012). Persistent inflammation and immunosuppression: A common syndrome and new horizon for surgical intensive care. J. Trauma Acute Care Surg..

[37] Decker D., Schondorf M., Bidlingmaier F., Hirner A., von Ruecker A.A. (1996). Surgical stress induces a shift in the type-1/type-2 T-helper cell balance, suggesting down-regulation of cell-mediated and up-regulation of antibody-mediated immunity commensurate to the trauma. Surgery.

[38] Baier P.K., Wolff-Vorbeck G., Eggstein S., Baumgartner U., Hopt U.T. (2005). Cytokine expression in colon carcinoma. Anticancer Res..

[39] Cusack B., Buggy D.J. (2020). Anaesthesia, analgesia, and the surgical stress response. BJA Educ..

[40] O’Leary D.P., Wang J.H., Cotter T.G., Redmond H.P. (2013). Less stress, more success? Oncological implications of surgery-induced oxidative stress. Gut.

[41] Stevens J.L., Feelisch M., Martin D.S. (2019). Perioperative oxidative stress: The unseen enemy. Anesth. Analg..

[42] Coffey J.C., Wang J.H., Smith M.J., Bouchier-Hayes D., Cotter T.G., Redmond H.P. (2003). Excisional surgery for cancer cure: Therapy at a cost. Lancet Oncol..

[43] Shibata J., Ishihara S., Tada N., Kawai K., Tsuno N.H., Yamaguchi H., Sunami E., Kitayama J., Watanabe T. (2015). Surgical stress response after colorectal resection: A comparison of robotic, laparoscopic, and open surgery. Tech. Coloproctol..

[44] Krzystek-Korpacka M., Zawadzki M., Szufnarowski K., Bednarz-Misa I., Gorska S., Witkiewicz W., Gamian A. (2018). The perioperative dynamics of IL-7 following robot-assisted and open colorectal surgery. Sci. Rep..

[45] Fleszar M.G., Fortuna P., Zawadzki M., Hodurek P., Bednarz-Misa I., Witkiewicz W., Krzystek-Korpacka M. (2021). Sex, Type of surgery, and surgical site infections are associated with perioperative cortisol in colorectal cancer patients. J. Clin. Med..

[46] Zawadzki M., Krzystek-Korpacka M., Gamian A., Witkiewicz W. (2017). Comparison of inflammatory responses following robotic and open colorectal surgery: A prospective study. Int. J. Color. Dis..

[47] Krzystek-Korpacka M., Zawadzki M., Lewandowska P., Szufnarowski K., Bednarz-Misa I., Jacyna K., Witkiewicz W., Gamian A. (2019). Distinct chemokine dynamics in early postoperative period after open and robotic colorectal surgery. J. Clin. Med..

[48] Fleszar M.G., Fortuna P., Zawadzki M., Kosak B., Krzystek-Korpacka M. (2020). Simultaneous LC-MS/MS-based quantification of free 3-nitro-L-tyrosine, 3-chloro-L-tyrosine, and 3-bromo-L-tyrosine in plasma of colorectal cancer patients during early postoperative period. Molecules.

[49] Bednarz-Misa I., Fleszar M.G., Zawadzki M., Kapturkiewicz B., Kubiak A., Neubauer K., Witkiewicz W., Krzystek-Korpacka M. (2020). L-Arginine/NO pathway metabolites in colorectal cancer: Relevance as disease biomarkers and predictors of adverse clinical outcomes following surgery. J. Clin. Med..

[50] Kim R. (2018). Effects of surgery and anesthetic choice on immunosuppression and cancer recurrence. J. Transl. Med..

[51] Zhang B., Xiao Q., Ma Q., Han L. (2023). Clinical treatment for persistent inflammation, immunosuppression and catabolism syndrome in patients with severe acute pancreatitis (Review). Exp. Ther. Med..

[52] Yin J., Chen Y., Huang J.L., Yan L., Kuang Z.S., Xue M.M., Sun S., Xiang H., Hu Y.Y., Dong Z.M. (2021). Prognosis-related classification and dynamic monitoring of immune status in patients with sepsis: A prospective observational study. World J. Emerg. Med..

[53] Horiguchi H., Loftus T.J., Hawkins R.B., Raymond S.L., Stortz J.A., Hollen M.K., Weiss B.P., Miller E.S., Bihorac A., Larson S.D. (2018). Sepsis and Critical Illness Research Center Investigators. Innate Immunity in the Persistent Inflammation, Immunosuppression, and Catabolism Syndrome and Its Implications for Therapy. Front. Immunol..

[54] Mira J.C., Brakenridge S.C., Moldawer L.L., Moore F.A. (2017). Persistent Inflammation, Immunosuppression and Catabolism Syndrome. Crit. Care Clin..

[55] Chadda K.R., Puthucheary Z. (2024). Persistent inflammation, immunosuppression, and catabolism syndrome (PICS): A review of definitions, potential therapies, and research priorities. Br. J. Anaesth..

[56] Forget P., Simonet O., De Kock M. (2013). Cancer surgery induces inflammation, immunosuppression and neo-angiogenesis, but is it influenced by analgesics?. F1000Research.

[57] Efron P.A., Mohr A.M., Bihorac A., Horiguchi H., Hollen M.K., Segal M.S., Baker H.V., Leeuwenburgh C., Moldawer L.L., Moore F.A. (2018). Persistent inflammation, immunosuppression, and catabolism and the development of chronic critical illness after surgery. Surgery.

[58] Li Q. (2023). Pituitary-immune bidirectional crosstalk under systemic inflammation. PLoS Biol..

[59] Schoenborn J.R., Wilson C.B. (2007). Regulation of interferon-gamma during innate and adaptive immune responses. Adv. Immunol..

[60] Quirant-Sánchez B., Plans-Galván O., Lucas E., Argudo E., Martinez-Cáceres E.M., Arméstar F. (2023). HLA-DR expression on monocytes and sepsis index are useful in predicting sepsis. Biomedicines.

[61] Tai L.H., de Souza C.T., Bélanger S., Ly L., Alkayyal A.A., Zhang J., Rintoul J.L., Ananth A.A., Lam T., Breitbach C.J. (2013). Preventing postoperative metastatic disease by inhibiting surgery-induced dysfunction in natural killer cells. Cancer Res..

[62] Konjević G.M., Vuletić A.M., Mirjačić Martinović K.M., Larsen A.K., Jurišić V.B. (2019). The role of cytokines in the regulation of NK cells in the tumor environment. Cytokine.

[63] Chen Y., Wang J., Liu C., Su L., Zhang D., Fan J., Yang Y., Xiao M., Xie J., Xu Y. (2020). IP-10 and MCP-1 as biomarkers associated with disease severity of COVID-19. Mol. Med..

[64] Dufour J.H., Dziejman M., Liu M.T., Leung J.H., Lane T.E., Luster A.D. (2002). IFN-gamma-inducible protein 10 (IP-10; CXCL10)-deficient mice reveal a role for IP-10 in effector T cell generation and trafficking. J. Immunol..

[65] Madhurantakam S., Lee Z.J., Naqvi A., Prasad S. (2023). Importance of IP-10 as a biomarker of host immune response: Critical perspective as a target for biosensing. Curr. Res. Biotechnol..

[66] Yu J., Wei M., Becknell B., Trotta R., Liu S., Boyd Z., Jaung M.S., Blaser B.W., Sun J., Benson D.M. (2006). Pro- and antiinflammatory cytokine signaling: Reciprocal antagonism regulates interferon-gamma production by human natural killer cells. Immunity.

[67] Krzystek-Korpacka M., Mierzchała-Pasierb M., Zawadzki M., Diakowska D., Witkiewicz W. (2021). Serum and erythrocyte antioxidant defense in colorectal cancer patients during early postoperative period: Potential modifiers and impact on clinical outcomes. Antioxidants.

[68] Amatya N., Garg A.V., Gaffen S.L. (2017). IL-17 Signaling: The Yin and the Yang. Trends Immunol..

[69] Mills K.H.G. (2023). IL-17 and IL-17-producing cells in protection versus pathology. Nat. Rev. Immunol..

[70] Mu X., Gu R., Tang M., Wu X., He W., Nie X. (2024). IL-17 in wound repair: Bridging acute and chronic responses. Cell Commun. Signal..

[71] Zhao J., Chen X., Herjan T., Li X. (2020). The role of interleukin-17 in tumor development and progression. J. Exp. Med..

[72] Amicarella F., Muraro M.G., Hirt C., Cremonesi E., Padovan E., Mele V., Governa V., Han J., Huber X., Droeser R.A. (2017). Dual role of tumour-infiltrating T helper 17 cells in human colorectal cancer. Gut.

[73] Briukhovetska D., Dörr J., Endres S., Libby P., Dinarello C.A., Kobold S. (2021). Interleukins in cancer: From biology to therapy. Nat. Rev. Cancer.

[74] Meier C., Brieger A. (2025). The role of IL-8 in cancer development and its impact on immunotherapy resistance. Eur. J. Cancer.

[75] Bazzichetto C., Milella M., Zampiva I., Simionato F., Amoreo C.A., Buglioni S., Pacelli C., Le Pera L., Colombo T., Bria E. (2022). Interleukin-8 in Colorectal Cancer: A Systematic Review and Meta-Analysis of Its Potential Role as a Prognostic Biomarker. Biomedicines..

[76] Wang L., Lan J., Tang J., Luo N. (2022). MCP-1 targeting: Shutting off an engine for tumor development (Review). Oncol. Lett..

[77] Lugano R., Ramachandran M., Dimberg A. (2020). Tumor angiogenesis: Causes, consequences, challenges and opportunities. Cell Mol. Life Sci..

[78] Liu Z.L., Chen H.H., Zheng L.L., Sun L.P., Shi L. (2023). Angiogenic signaling pathways and anti-angiogenic therapy for cancer. Signal Transduct. Target. Ther..

[79] Kong B., Michalski C.W., Friess H., Kleeff J. (2010). Surgical procedure as an inducer of tumor angiogenesis. Exp. Oncol..

[80] Shi Z., Yao C., Shui Y., Li S., Yan H. (2023). Research progress on the mechanism of angiogenesis in wound repair and regeneration. Front. Physiol..

[81] Selvaraj V., Sekaran S., Rajamani Sekar S.K. (2023). Surgical intervention as a driver of new angiogenesis in tumors-time to consider minimally invasive surgeries?. Int. J. Surg..

[82] Chen J., Gong C., Mao H., Li Z., Fang Z., Chen Q., Lin M., Jiang X., Hu Y., Wang W. (2018). E2F1/SP3/STAT6 axis is required for IL-4-induced epithelial-mesenchymal transition of colorectal cancer cells. Int. J. Oncol..

[83] Bednarz-Misa I., Diakowska D., Szczuka I., Fortuna P., Kubiak A., Rosińczuk J., Krzystek-Korpacka M. (2020). Interleukins 4 and 13 and their receptors are differently expressed in gastrointestinal tract cancers, depending on the anatomical site and disease advancement, and improve colon cancer cell viability and motility. Cancers.

[84] Chen H.M., Hung P.Y., Chen C.H., Yu Y.J., Syu M.S., Hu M.C. (2022). HSD3B1 expression is upregulated by interleukin 4 in HT-29 colon cancer cells via multiple signaling pathways. Int. J. Mol. Sci..

[85] Li J., Huang L., Zhao H., Yan Y., Lu J. (2020). The role of interleukins in colorectal cancer. Int. J. Biol. Sci..

[86] Song X., Traub B., Shi J., Kornmann M. (2021). Possible roles of interleukin-4 and -13 and their receptors in gastric and colon cancer. Int. J. Mol. Sci..

[87] Ito S.E., Shirota H., Kasahara Y., Saijo K., Ishioka C. (2017). IL-4 blockade alters the tumor microenvironment and augments the response to cancer immunotherapy in a mouse model. Cancer Immunol. Immunother..

[88] Lopez-Perez D., Prados-Lopez B., Galvez J., Leon J., Carazo A. (2024). Eosinophils in colorectal cancer: Emerging insights into anti-tumoral mechanisms and clinical implications. Int. J. Mol. Sci..

[89] Li B., Wang F., Ma C., Hao T., Geng L., Jiang H. (2019). Predictive value of IL-18 and IL-10 in the prognosis of patients with colorectal cancer. Oncol. Lett..

[90] Kouro T., Takatsu K. (2009). IL-5- and eosinophil-mediated inflammation: From discovery to therapy. Int. Immunol..

[91] Saraiva A.L., Carneiro F. (2018). New insights into the role of tissue eosinophils in the progression of colorectal cancer: A literature review. Acta Med. Port..

[92] Karagiannidis I., Salataj E., Said Abu Egal E., Beswick E.J. (2021). G-CSF in tumors: Aggressiveness, tumor microenvironment and immune cell regulation. Cytokine.

[93] O’Rourke K., Huddart S. (2020). Surgical stress response and cancer outcomes: A narrative review. Dig. Med. Res..

[94] Jin K., Qian C., Lin J., Liu B. (2023). Cyclooxygenase-2-prostaglandin E2 pathway: A key player in tumor-associated immune cells. Front. Oncol..

[95] Sheng J., Sun H., Yu F.-B., Li B., Zhang Y., Zhu Y.-T. (2020). The Role of Cyclooxygenase-2 in colorectal cancer. Int. J. Med. Sci..

[96] Tong D., Liu Q., Wang L.A., Xie Q., Pang J., Huang Y., Wang L., Liu G., Zhang D., Lan W. (2018). The roles of the COX2/PGE2/EP axis in therapeutic resistance. Cancer Metastasis Rev..

[97] Glasner A., Avraham R., Rosenne E., Benish M., Zmora O., Shemer S., Meiboom H., Ben-Eliyahu S. (2010). Improving survival rates in two models of spontaneous postoperative metastasis in mice by combined administration of a beta-adrenergic antagonist and a cyclooxygenase-2 inhibitor. J. Immunol..

[98] Wei J., Zhang J., Wang D., Cen B., Lang J.D., DuBois R.N. (2022). The COX-2-PGE2 pathway promotes tumor evasion in colorectal adenomas. Cancer Prev. Res..

[99] Kulesza A., Paczek L., Burdzinska A. (2023). The role of COX-2 and PGE2 in the regulation of immunomodulation and other functions of mesenchymal stromal cells. Biomedicines.

[100] Wang Y.J., Xie X.L., Liu H.Q., Tian H., Jiang X.Y., Zhang J.N., Chen S.X., Liu T., Wang S.L., Zhou X. (2023). Prostaglandin F2α synthase promotes oxaliplatin resistance in colorectal cancer through prostaglandin F2α-dependent and F2α-independent mechanism. World J. Gastroenterol..

[101] Qualtrough D., Kaidi A., Chell S., Jabbour H.N., Williams A.C., Paraskeva C. (2007). Prostaglandin F(2alpha) stimulates motility and invasion in colorectal tumor cells. Int. J. Cancer.

[102] Kidd P. (2003). Th1/Th2 Balance: The Hypothesis, its Limitations, and Implications for Health and Disease. Altern. Med. Rev..

[103] Bezu L., Öksüz A.D., Bell M., Buggy D., Diaz-Cambronero O., Enlund M., Forget P., Gupta A., Hollmann M.W., Ionescu D. (2024). Perioperative immunosuppressive factors during cancer surgery: An updated review. Cancers.

[104] Krzystek-Korpacka M., Fleszar M.G., Fortuna P., Gostomska-Pampuch K., Lewandowski Ł., Piasecki T., Kosyk B., Szeląg A., Trocha M. (2021). Modulation of prostanoids profile and counter-regulation of SDF-1α/CXCR4 and VIP/VPAC2 expression by sitagliptin in non-diabetic rat model of hepatic ischemia-reperfusion injury. Int. J. Mol. Sci..

